# A Brain without Brakes: Reduced Inhibition Is Associated with Enhanced but Dysregulated Plasticity in the Aged Rat Auditory Cortex

**DOI:** 10.1523/ENEURO.0051-18.2018

**Published:** 2018-09-05

**Authors:** J. Miguel Cisneros-Franco, Lydia Ouellet, Brishna Kamal, Etienne de Villers-Sidani

**Affiliations:** 1 Department of Neurology and Neurosurgery, Montreal Neurological Institute, McGill University, Montreal QC H3A 2B4, Canada; 2 Centre for Research on Brain, Language, and Music, Montreal QC H3G 2A8, Canada

**Keywords:** Aging, auditory cortex, cortical plasticity, GABA, inhibition, training

## Abstract

During early developmental windows known as critical periods (CPs) of plasticity, passive alterations in the quality and quantity of sensory inputs are sufficient to induce profound and long-lasting distortions in cortical sensory representations. With CP closure, those representations are stabilized, a process requiring the maturation of inhibitory networks and the maintenance of sufficient GABAergic tone in the cortex. In humans and rodents, however, cortical inhibition progressively decreases with advancing age, raising the possibility that the regulation of plasticity could be altered in older individuals. Here we tested the hypothesis that aging results in a destabilization of sensory representations and maladaptive dysregulated plasticity in the rat primary auditory cortex (A1). Consistent with this idea, we found that passive tone exposure is sufficient to distort frequency tuning in the A1 of older but not younger adult rats. However, we also found that these passive distortions decayed rapidly, indicating an ongoing instability of A1 tuning in the aging cortex. These changes were associated with a decrease in GABA neurotransmitter concentration and a reduction in parvalbumin and perineuronal net expression in the cortex. Finally, we show that artificially increasing GABA tone in the aging A1 is sufficient to restore representational stability and improve the retention of learning.

## Significance Statement

In this study, we examined brain plasticity in the auditory cortex of young adult and older adult rats in the context of different types of auditory stimulation and training. Surprisingly, older brains retained an equal or even higher potential for plasticity compared to young adults. In older brains, however, changes elicited by auditory stimulation and training were rapidly lost, suggesting that such increased plasticity might be detrimental, as the older brains were unable to consolidate these changes. This increased but poorly regulated plasticity was associated with a reduction in cortical inhibition, which normally maintains the stability of sensory representations in the young adult brain. Importantly, increasing inhibition artificially with clinically available drugs restored stability and improved the retention of learning.

## Introduction

During early developmental epochs known as critical periods (CPs) of plasticity, passive exposure to environmental sounds profoundly shapes frequency tuning in the rat primary auditory cortex (A1; Hensch, 2005; de Villers-Sidani et al., 2007). On closure of the CP, these experience-dependent alterations are consolidated, and A1 tuning becomes relatively resistant to passive sound exposures. CP closure in sensory cortices is associated with the maturation of functional and structural inhibitory elements (Hensch, 2005; Fritschy and Panzanelli, 2014), including the maturation of parvalbumin positive (PV^+^) interneurons (Kuhlman et al., 2013) and perineuronal nets (PNN; Wang and Fawcett, 2012). In the adult brain, plastic changes of the magnitude observed in the CP can be induced by down-regulating cortical inhibition (Fagiolini and Hensch, 2000) or disrupting elements involved in the stabilization of cortical representations such as PNNs (Pizzorusso et al., 2002; Carulli et al., 2010; Wang and Fawcett, 2012). Plastic changes of this magnitude otherwise still occur in the mature brain, but regulation mechanisms restrict them mainly to the context of behavior (Blake et al., 2006; Polley et al., 2006; Caroni et al., 2012). This tight gating of plasticity and resulting relative stability in the mature brain contribute to the consolidation and retention of new perceptuo-motor skills acquired through learning (Maffei and Turrigiano, 2008; Caroni et al., 2012).

Cortical inhibitory circuits are almost invariably affected by natural aging, as evidenced by a reduction of inhibitory tone and specific inhibitory interneurons such as PV^+^ and somatostatin-positive (SST^+^) cells in older brains (Caspary et al., 2008; Stanley et al., 2012; Ouellet and de Villers-Sidani, 2014). Given the importance of inhibitory processes in the regulation of plasticity and learning, it is reasonable to speculate that aging could have a significant impact on the mechanisms of learning in the brain (Caspary et al., 2008; Liguz-Lecznar et al., 2014). Loss of inhibition could lead to a state of cortical instability where sensory representations are easily distorted by nonspecific passive experiences, as is the case with the CP (Zhou et al., 2011). Such impairments could likely explain the noisy sensory processing and less effective learning and recovery observed in older rodents (Liguz-Lecznar et al., 2014) and humans (Boyke et al., 2008; Knoflach et al., 2012). Here, we tested these ideas using a combination of controlled passive pure tone exposure, pharmacological experiments, and behavioral training in young and old rats. We found that experience-dependent plasticity is paradoxically enhanced but unstable in old rats compared to young controls. Such instability was found even for relatively short 10 minute-long exposures and was paralleled by a reduction in the number of PV^+^ cells and PNNs. Finally, we also demonstrated that this instability is associated with a more rapid decay of learning that can be reversed by artificially enhancing GABA tone in the brain.

## Materials and Methods

All experimental procedures used in this study were approved by the Animal Care Committee and follow established guidelines. Twenty-three immature Long-Evans rats of either sex [age postnatal 10 days (P10) to P24], 24 Long-Evans young adult rats of either sex (age 6–8 mo), and 28 Long-Evans old adult rats of either sex (22–24 mo) were used for this study.

### Passive sound exposure

The tone pip-exposed rats were housed for 1 or 2 consecutive weeks (24 h/d, 7 d/wk) in a sound attenuated chamber equipped with a speaker. The exposure sequences were generated using custom Matlab routines and contained repetitive trains of six 25-ms-long 5- or 10-kHz pips with 5-ms cosine gates presented at a rate of 5 p.p.s. at an intensity of 70 dB SPL.

### Mapping the auditory cortex

For A1 mapping, the rats were premedicated with dexamethasone (0.2 mg/kg) to minimize brain edema. They were anesthetized with ketamine/xylazine/acepromazine (65/13/1.5 mg/kg, i.p.) followed by a continuous delivery of isoflurane 1% in oxygen delivered via endotracheal intubation and mechanical ventilation. Vital signs were monitored using a MouseOx device (Starr Life Sciences). Body temperature was monitored with a rectal probe and maintained at 37°C with a homeothermic blanket system. The rats were held by the orbits in a custom-designed head holder, leaving the ears unobstructed. The cisterna magna was drained of cerebrospinal fluid to further minimize brain edema. The left temporalis muscle was reflected, auditory cortex (AC) was exposed, and the dura was resected. The cortex was maintained under a thin layer of silicone oil to prevent desiccation.

Cortical responses were recorded with 32–64-channel tungsten microelectrode arrays (Neuronexus). The microelectrode array was lowered orthogonally into the cortex to a depth of 470–600 µm (layers 4/5) where vigorous stimulus-driven responses were obtained. The extracellular neural action potentials were amplified, filtered (0.3–5 kHz), sorted, and monitored on-line. Acoustic stimuli were generated using TDT System III (Tucker-Davis Technologies) and delivered in a free-field manner to the right ear through a calibrated speaker (Tucker-Davis Technologies). A software package (OpenEx; Tucker-Davis Technologies) was used to generate acoustic stimuli, monitor cortical response properties on-line, and store data for off-line analysis. The evoked spikes of a single neuron or a small cluster of neurons were collected at each site.

Frequency-intensity receptive fields were reconstructed by presenting pure tones of 63 frequencies (1–48 kHz; 0.1-octave increments; 25-ms duration; 5-ms ramps) at 8 sound intensities (0–70 dB SPL in 10-dB increments) to the contralateral ear at a rate of one stimulus per second. Ten-minute-long trains of 50-ms tone pips were presented at 3 pulses per second at a sound intensity of 70 dB SPL. Each train had a commonly occurring frequency (standard) with a probability of occurrence of 80% and 5 pseudo-randomly distributed oddball frequencies presented 20% of the time with no repetition. The oddball frequencies in the train had a constant separation of 1 octave.

### Electrophysiological data analysis

The characteristic frequency (CF) of a cortical site was defined as the frequency at the tip of the V-shaped tuning curve. For flat-peaked tuning curves, the CF was defined as the midpoint of the plateau at threshold. For tuning curves with multiple peaks, the CF was defined as the frequency at the most sensitive tip (i.e., with lowest threshold). The CF and threshold were determined using an automated routine developed in the Matlab environment (The MathWorks).

To generate A1 maps, Voronoi tessellation (a Matlab routine) was performed to create tessellated polygons with electrode penetration sites at their centers. Each polygon was assigned the characteristics (i.e., CF) of the corresponding penetration site. In this way, every point on the surface of the AC was linked to the characteristics experimentally derived from its closest sampled cortical site. Primary AC (A1) was identified based on its rostral-to-caudal tonotopy, reliable short-latency tone-evoked neuronal responses, and relatively sharp V-shaped RF. To examine A1 map plasticity, we compared the percentage of A1 sites with CFs in 12 bins (width = ½ octave) spanning the spectrum of presented tones.

Normalized responses to standard and oddball tones were obtained by dividing the average firing rate recorded in the 50 ms after the occurrence of each tone presentation by the average firing rate observed during the 50 ms after the first standard or oddball tone in the sequence. Asymptotes for standard and oddball responses were calculated by fitting exponential functions with a least squares method to the normalized response data from each recorded neuron. Simple linear regression of the normalized responses to the standard tone for the interval from event no. 150 to event no. 1200 was performed. The slope of the resulting best fit line was computed to determine the level of adaptation for each recorded site.

### Training

Behavior was shaped in three phases. During the first phase, rats were trained to make a nose poke response to obtain a food reward. During the second phase, rats were trained to make a nose poke only after presentation of an auditory stimulus. During the third phase, the actual training program, rats were trained to make a nose poke only for the target stimulus (a 5-kHz pure tone) and not for a foil nontarget stimulus (10-kHz pure tone). The tones were presented at 60 dB SPL, stimulus presentation was randomized, and the probability of a target stimulus presentation was set at 20%. Training was performed in an acoustically transparent operant training chamber (60 × 45 × 35 cm, length × width × height) contained within a sound-attenuated chamber. Sound presentation and response recording were performed using OpenEx software and RZ6 auditory processing hardware from Tucker-Davis Technology and delivered in a free field manner through a calibrated loudspeaker.

The intertrial interval was selected at random from a range of 4–6 s. A rat’s behavioral state at any point in time was classified as either “go” (producing a nose poke behavior) or “no-go.” For a given trial, the rat could elicit one of four reinforcements produced by the combinations of responses (go or no-go) and stimulus properties (target or nontarget). Go responses within 5 s of a target were scored as a hit; a failure to respond within this time window was scored as a miss; a go response within 5 s of a nontarget stimulus was scored as a false positive; the absence of a response was scored as a withhold. A hit triggered the delivery of a food pellet. A miss or false positive initiated a 5-s time-out period during which time the house lights were turned off and no stimuli were presented. A withhold did not produce a reward or a time-out. Psychometric functions and stimulus target recognition indexes (D-prime) were calculated for each training session by plotting the percentage of go responses as a function of the total number of target stimuli (i.e., hit ratio) and the percentage of false positives as a function of the total number of foils (i.e., false-positive ratio). Learning curves were reconstructed by plotting the D-prime measure reached over successive days of training.

### GABA microdialysis

Immediately after craniotomy (see Methods: Electrophysiology), a microdialysis probe (CMA 12 Microdialysis probe, Harvard Apparatus) was implanted in the AC using the stereotaxic coordinates (Paxinos and Watson, 2007): bregma AP, –4.5 mm; ML, –7 mm; DV, 4.5 mm. The pump rate was set at 0.09 mL/h (PHD ultra 4400 Syringe pump, Harvard Apparatus). Samples were manually collected and frozen at –80°C until analysis with high performance liquid chromatography (Reinhoud et al., 2013).

### Immunohistochemistry

Immediately following the end of recording sessions, rats received a high dose of pentobarbital (85 mg/kg i.p.) and were perfused intracardially with 4% paraformaldehyde in 0.1 m PBS at pH 7.2. Immediately after perfusion, rat brains were removed and placed in the same fixative overnight for further fixation and then transferred to a 30% sucrose solution, snap-frozen, and stored at –80°C until sectioning. Fixed material was cut in the coronal plane along the tonotopic axis of A1 on a freezing microtome at 40 µm. Tissue was incubated overnight at 4°C in either monoclonal or polyclonal antisera (for anti-PV: #P-3088, dilution 1:10,000, Sigma-Aldrich; for PNN, fluorescein wisteria floribunda lectin #FL-1351, dilution 1:200, Vector Laboratories). Tissue samples were always processed in pairs during immunostaining procedures to limit variables relative to antibody penetration, incubation time, and post-sectioning age/condition of tissue. A Zeiss LSM 510 Meta confocal microscope was used to assess fluorescence in the immunostained sections. Quantification of PV^+^ cells and PNN optical density was performed in ImageJ (National Institutes of Health) and MetaMorph imaging software (Molecular Devices Systems), respectively. Digital images of A1 cortical sections were taken with a 40× objective (Zeiss LSM 510). All quantification was assessed in 300–400-µm-wide A1 sectors (rostral, middle, caudal) extending from layer 1 to the underlying white matter by an experimenter blind to the age of the animals. PV^+^ cells were classified into four subclasses as follows: low-PV, 0–0.8 × 10^5^; intermediate low-PV, 8–1.6 × 10^5^; intermediate high-PV, 1.6–2.4 × 10^5^; high-PV, >2.4 × 10^5^. PNNs were classified into four subclasses as follows: low PV, 0–1 × 10^4^, intermediate low-PV, 1–2 × 10^5^; intermediate high-PV, 2–3 × 10^5^; high-PV, >3 × 10^5^.

### Statistical analysis

For normally distributed data, statistical significance was assessed using unpaired two-tailed *t* tests or two-way analysis of variance with Tukey *post hoc* correction for multiple comparisons. Wilcoxon rank-sum test or Kruskal–Wallis test with Tukey *post hoc* correction for multiple comparisons were used for nonparametric data analysis. Data are presented as mean ± SEM or median ± median absolute deviation (MAD). Superscript letters listed with *p*-values correspond to the statistical tests shown in Table 1.

**Table 1.** Statistical table.

	Data structure	Type of test	Statistic and *p* value
a	Normal distribution	2-way ANOVA; Tukey–Kramer test	*F*(11,168) = 14.84, *p* < 0.001; *p* < 0.001
b	Normal distribution	2-way ANOVA; Tukey–Kramer test	*F*(11,72) = 4.02, *p* < 0.001; *p* = 0.87
c	Normal distribution	2-way ANOVA; Tukey–Kramer test	*F*(11,72) = 10.77, *p* < 0.001; *p* < 0.001
d	Normal distribution	2-way ANOVA; Tukey–Kramer test	*F*(11,72) = 13.13, *p* < 0.001; *p* < 0.001, *p* = 0.35
e	Normal distribution	2-way ANOVA; Tukey–Kramer test	*F*(11,72) = 2.69, *p* = 0.005; *p* = 1, *p* = 0.96
f	Normal distribution	2-way ANOVA; Tukey–Kramer test	*F*(11,72) = 7.23, *p* < 0.001; *p* = 1, *p* < 0.001
g	Normal distribution	2-way ANOVA; Tukey–Kramer test	*F*(11,132) = 14.62, *p* = 0; *p* < 0.001, *p* = 0.15
h	Normal distribution	2-way ANOVA; Tukey–Kramer test	*F*(11,120) = 12.58, *p* = 0; *p* < 0.001, *p* = 0.1
i	Nonnormal distribution	Wilcoxon rank-sum test	*z* = –4.099, *p* = 4.1 × 10^–5^
j	Nonnormal distribution	Wilcoxon rank-sum test	*z* = –3.187, *p* = 0.0014
K	Normal distribution	*t* test	*t*(579) = 5.64, *p* < 0.001
l	Normal distribution	*t* test	*t*(395) = 3.35, *p* = 9 × 10^–4^
m	Normal distribution	*t* test	*t*(750) = 0.75, *p* = 0.45
*n*	Normal distribution	*t* test	*t*(408) = 0.64, *p* = 0.52
o	Normal distribution	*t* test	*t*(383) = 2.55, *p* = 0.011
*p*	Nonnormal distribution	Wilcoxon rank-sum test	*z* = –4.4, *p* = 1.1 × 10^–5^
q	Nonnormal distribution	Wilcoxon rank-sum test	*z* = ×2.46, *p* = 0.013
r	Normal distribution	*t* test	*t*(720) = 5.29, *p* < 0.001
s	Normal distribution	*t* test	*t*(345) = 2.1, *p* = 0.03
*t*	Normal distribution	*t* test	*t*(690) = 0.86, *p* = 0.39
u	Normal distribution	*t* test	*t*(308) = 0.08, *p* = 0.94
v	Normal distribution	*t* test	*t*(18) = 2.32, *p* = 0.032
w	Normal distribution	2-way ANOVA; Tukey–Kramer test	*F*(11,72) = 13.42, *p* < 0.001; *p* = 0.018
x	Normal distribution	2-way ANOVA; Tukey–Kramer test	*F*(11,72) = 6.57, *p* < 0.001; *p* = 0.004, *p* = 0.41
y	Normal distribution	*t* test	*t*(6) = 5.02, *p* = 0.002
*z*	Normal distribution	2-way ANOVA; Tukey–Kramer test	*F*(11,72) = 6.68, *p* < 0.001; *p* = 0.01
ab	Normal distribution	2-way ANOVA; Tukey–Kramer test	*F*(11,72) = 1.41, *p* = 0.18
ac	Normal distribution	2-way ANOVA; Tukey–Kramer test	*F*(11,72) = 5.42, *p* < 0.001; *p* = 0.022
ad	Normal distribution	*t* test	*t*(6) = 2.53, *p* = 0.04
ae	Normal distribution	*t* test	*t*(6) = 3.66, *p* = 0.01
af	Nonnormal distribution	Kruskal–Wallis test; Tukey–Kramer *post hoc* test	*H*(4) = 14.52, *p* = 0.0058; *p* = 0.52, *p* = 0.011, *p* = 0.96, *p* = 0.97
ag	Nonnormal distribution	Kruskal–Wallis test; Tukey–Kramer *post hoc* test	*H*(4) = 83.97, *p* < 0.0001; *p* < 0.0001, *p* < 0.001, *p* = 0.96, *p* = 0.003
ah	Nonnormal distribution	Kruskal–Wallis test; Tukey–Kramer *post hoc* test	*H*(4) = 13, *p* = 0.011; *p* = 0.82, *p* = 0.005, *p* = 0.99, *p* = 0.99
ai	Nonnormal distribution	Kruskal–Wallis test; Tukey–Kramer *post hoc* test	*H*(4) = 17.24, *p* = 0.0017; *p* = 0.48, *p* = 0.04, *p* = 0.004, *p* = 0.8, *p* = 0.99
aj	Nonnormal distribution	Kruskal–Wallis test; Tukey–Kramer *post hoc* test	*H*(4) = 22.06, *p* < 0.001; *p* = 0.004, *p* = 0.039, *p* = 0.99, *p* = 0.85

## Results

### Passive tone exposure induces significant shifts in A1 tuning

**Figure 1.**
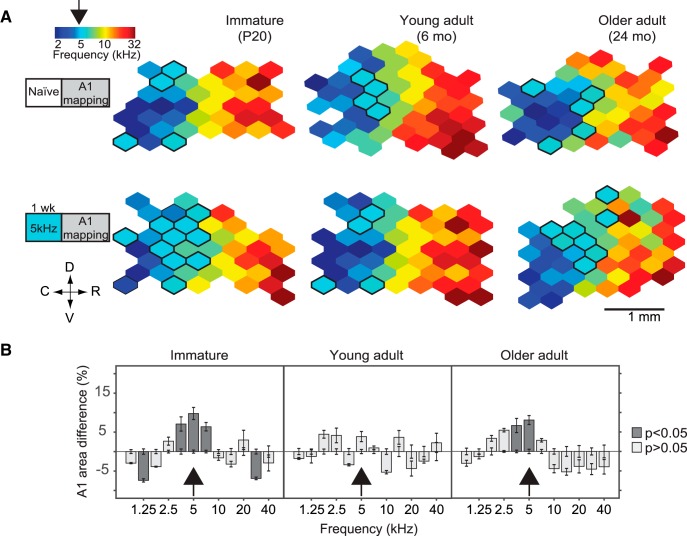
Passive sound exposure alters frequency tuning in the aged A1. ***A***, Representative A1 CF maps from naïve rats (top) and from rats exposed to 5-kHz pure tones during 1 week (bottom). D, dorsal; C, caudal; R, rostral; V, ventral. ***B***, Difference in frequency tuning between naïve and exposed rats expressed as A1 percentage area and separated by CF for immature, young adult, and old adult groups. Immature group: *n* = 8, recorded sites = 389; YA: *n* = 8, recorded sites = 403; OA: *n* = 8, recorded sites = 382; immature-exposed: *n* = 8, recorded sites = 362; YA-exposed: *n* = 4, recorded sites = 177; OA-exposed: *n* = 4, recorded sites = 168. Values shown are mean, two-way ANOVA with Tukey–Kramer correction.

Aging is characterized by a progressive reduction in cortical inhibition to levels akin to those observed during developmental critical periods (Caspary et al., 2008; Stanley et al., 2012; Ouellet and de Villers-Sidani, 2014; Stebbings et al., 2016). This raises the possibility that the old brain has in fact a higher plastic potential than its young adult counterpart. To test this hypothesis, we examined the effect of pure-tone exposure on spectral tuning in the aged A1. We exposed old adult rats (OA, 22–23 months old, *n* = 4) to 5 kHz tone pips for 1 week. For comparison, the same exposure was used in young adult (YA, 6–8 months old, *n* = 4) and immature rats in their CP window (I, P10–P17, *n* = 8; Fig. 1*A*). We then compared the proportion of A1 neurons whose characteristic frequency was close to the exposure frequency. As expected, there was a clear effect of 5-kHz tone exposure on the CF of immature rats (two-way ANOVA, exposure group × frequency bin, *F*(11,168) = 14.84, *p* < 0.001^a^). Such an exposure resulted in a significant overrepresentation of the exposure tone in A1 of immature rats (average % difference in the proportion of recording sites tuned within ½ octave of exposure tone, relative to control: 9.77 ± 1.54% increase, *p* < 0.001^a^, with Tukey–Kramer correction) but not in the young adult group (*F*(11,72) = 4.02, *p* < 0.001; 3.84 ± 1.3% increase, *p* = 0.87^b^, with Tukey–Kramer correction; Fig. 1B). Passive tone exposure, however, resulted in a significant overrepresentation of the exposure tone in the aged A1 group (*F*(11,72) = 10.77, *p* < 0.001, two-way ANOVA; 8.05 ± 1.14% increase, *p* < 0.001^c^, with Tukey–Kramer correction).

**Figure 2.**
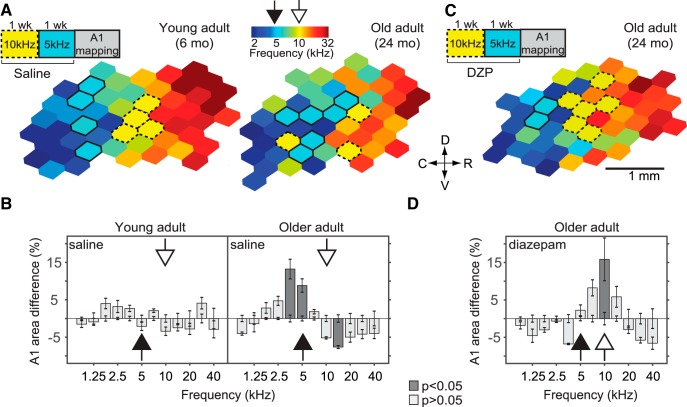
Restoration of inhibition stabilizes frequency representation in the aged A1. Young and old adult rats were exposed to 10-kHz pure tones for 1 week, followed immediately by exposure to 5-kHz pure tones for 1 week. ***A***, Representative A1 CF maps from young (left) and old (right) adult rats that received sham (saline) intraperitoneal injections during the 2-week passive exposure period. ***B***, Difference in frequency tuning between naïve and saline-treated rats expressed as A1 percentage area and separated by CF. ***C***, Representative A1 CF map from an old adult rat that received diazepam (DZP) intraperitoneal injections during the 2-week passive exposure period. ***D***, Difference in frequency tuning between naïve and DZP-treated rats. To investigate whether sequential exposure to pure tones would have a similar effect in immature rats, 2-week exposures were conducted starting on P10 as described in Fig. 2-1. YA-saline group: *n* = 4, recorded sites = 230; OA-saline: *n* = 4, recorded sites = 203; OA-diazepam: *n* = 4; recorded sites = 218. Values shown are mean, two-way ANOVA with Tukey–Kramer correction. Conventions as in Fig. 1.

To further document the extent of this tuning instability in older rats, we examined the effect of two consecutive pure-tone exposures over a 2-week period. Young (*n* = 4) and old (*n* = 4) adult rats were exposed to 10-kHz tone pips during the first week and to 5-kHz tone pips during the second week (Fig. 2*A*). This passive exposure protocol resulted in an overrepresentation of the second (5-kHz) exposure tone in A1 of aged rats (*F*(11,72) = 13.13, *p* < 0.001, two-way ANOVA; 5 kHz: 8.81 ± 1.7% increase, *p* < 0.001; 10 kHz: 5.18 ± 1.3% decrease, *p* = 0.35^d^, with Tukey–Kramer correction) but did not alter the frequency tuning map of young adults (*F*(11,72) = 2.69, *p* = 0.005, two-way ANOVA; 5 kHz: 2.1 ± 0.56% decrease, *p* = 1; 10 kHz: 3.4 ± 0.57% decrease, *p* = 0.96^e^, with Tukey–Kramer correction; Fig. 2*B*).

10.1523/ENEURO.0051-18.2018.f2-1 Figure 2-1 Immature rats exposed sequentially to pure tones over 2 weeks show an overrepresentation of the first tone of exposure. Rats were exposed starting at P10 to 5-kHz pure tones for 1 week, followed immediately by exposure to 10-kHz pure tones for 1 week. ***A***, Representative A1 CF maps from rats that received sham (saline, left) or diazepam (right) intraperitoneal injections during the 2-week passive exposure period. ***B***, Difference in frequency tuning between naïve and treated rats expressed as A1 percentage area and separated by CF. Values shown are mean ± SEM; two-way ANOVA corrected for multiple comparisons using Tukey–Kramer test. Download Figure 2-1, EPS file.

### Enhancing cortical inhibition stabilizes frequency representation in the aged A1

Having documented the existence of age-related tuning instability in A1, and considering the reduction of intracortical inhibition in sensory cortices associated with advanced age (Lehmann et al., 2012; Wang and Fawcett, 2012), we hypothesized that increasing GABAergic tone would restore the excitatory/inhibitory (E/I) balance and prevent further plastic changes elicited by passive tone exposure. To determine the effect of enhancing inhibition on frequency tuning stability in aged rats, we systemically administered the GABA_A_ agonist diazepam (1 mg/kg i.p., twice a day; *n* = 4) during two consecutive pure-tone exposures as described above (10-kHz pure tones for 1 week followed by 5-kHz pure tones for 1 week; Fig. 2*C*). We found that diazepam administration resulted in an overrepresentation of the first (10-kHz) rather than the second (5-kHz) exposure tone (*F*(11,72) = 7.23, *p* < 0.001, two-way ANOVA; 5 kHz: 2.23 ± 1.45% increase, *p* = 1; 10 kHz: 13.8 ± 5.7% increase, *p* < 0.001^f^, corrected with Tukey–Kramer test; Fig. 2*D*).

To investigate whether sequential exposure to pure tones would have a similar effect in immature animals as in old adult animals, we used a sequential 2-week exposure paradigm starting at P10 (5 kHz pure tones for 1 week, followed by 10 kHz pure tones for 1 week, *n* = 5). Previous experiments have shown that passive tone exposure outside the CP for frequency tuning (∼P10–P14) does not alter the A1 tonotopic map (de Villers-Sidani et al., 2007). For this reason, we predicted that such an exposure would result in an overrepresentation of the tone presented during the CP—the first exposure tone—regardless of any subsequent tone presentation. As expected, and in contrast to the results observed in the OA group, we observed plasticity in response to the first exposure tone (*F*(11,132) = 14.62, *p* = 0, two-way ANOVA; 5 kHz: 13.27 ± 3.4% increase, *p* < 0.001; 10 kHz: 5.72 ± 1.6% decrease, *p* = 0.15^g^, corrected with Tukey–Kramer test; Fig. 2-1*A*, left) in the vehicle (saline) condition.

Although treatment with diazepam accelerates the closing of the CP (Iwai et al., 2003), it does not prevent experience-dependent plasticity from taking place (Hensch et al., 1998; Fagiolini and Hensch, 2000). In line with these observations, sequential tone exposure in immature rats treated with diazepam < (n = 4) resulted in a significant expansion of the tone presented during the span of the CP; i.e., the first exposure tone (*F*(11,120) = 12.58, *p* < 0.001, two-way ANOVA; 5 kHz: 11.93 ± 3.6% increase, *p* = 0.018; 10 kHz: 6.11 ± 1.1% decrease, *p* = 0.1^h^, corrected with Tukey–Kramer test; Fig. 2-1*A*, *B*, right).

### Reversal of adaptation in the immature and aged A1

**Figure 3.**
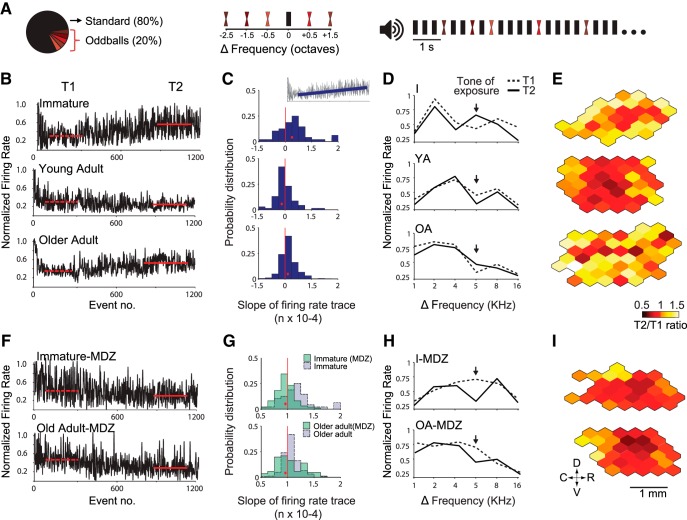
Improved adaptation in the immature and aged A1 following administration of the GABAA agonist midazolam. ***A***, Stimulation paradigm. Left, a standard (high-probability) tone was presented 80% of times. Five oddball (low-probability) tones distributed around the standard frequency (middle) were interspersed in the repetitive tone presentation (right). ***B***, Representative normalized responses of individual A1 neurons to a standard tone (5 or 12 kHz at a repetition rate of 3 Hz) as function of tone position in the stimulus sequence. Red horizontal lines represent the average normalized firing rate in response to the standard tone during two different intervals in the stimulus sequence: early (T1, event 100–300; dashed line), and late (T2, event 900–1100; solid line). Note that adaptation is reduced in both immature (I) and old adult rats. ***C***, Probability distribution plot of the slope of firing rate trace in response to the standard tone (interval from event no. 150–1200). Red dots denote the location of the median value for each group. Fig. 3-1 provides a summary of data related to adaptation in response to repetitive tones for all five groups. ***D***, Frequency tuning of representative A1 neurons during T1 (dashed line) and T2 (solid line). The normalized spike rate is plotted for the standard tone (arrow) and each of the five deviant tones. Note the acute change in tuning after standard-oddball presentation in I and OA rats. ***E***, Representative A1 activity maps depicting the change in firing rate at T2 relative to T1 (T2/T1 ratio of normalized firing rate). Warmer colors (white, yellow) denote neurons with reduced adaptation, notably in the I and OA groups. Same conventions apply for panels ***F–I***, which show that midazolam improved adaptation and prevented changes in tuning in the immature and aged A1. Immature group: *n* = 8, recorded sites = 376; YA: *n* = 4, recorded sites = 205; OA: *n* = 4, recorded sites = 192; I-MDZ: *n* = 8, recorded sites = 346; OA-MDZ: *n* = 4, recorded sites = 155.

Auditory neurons continuously monitor the environment, suppressing their response to repetitive sounds and making novel stimuli more salient (Ulanovsky et al., 2003; Malmierca et al., 2014). In the adult A1, such stimulus-specific adaptation prevents the overrepresentation of repetitive stimuli that drive plasticity during early development (Norena et al., 2006) and is also involved in the selection of A1 representations that should be selectively suppressed in the context of training (Froemke et al., 2013). With aging, however, receptive fields become less reliable across successive repetitions of the same set of stimuli (Turner et al., 2005). To examine the extent to which aging A1 neurons exhibit SSA, we used 10-min-long trains of pure tones (Fig. 3*A*).

**Figure 4.**
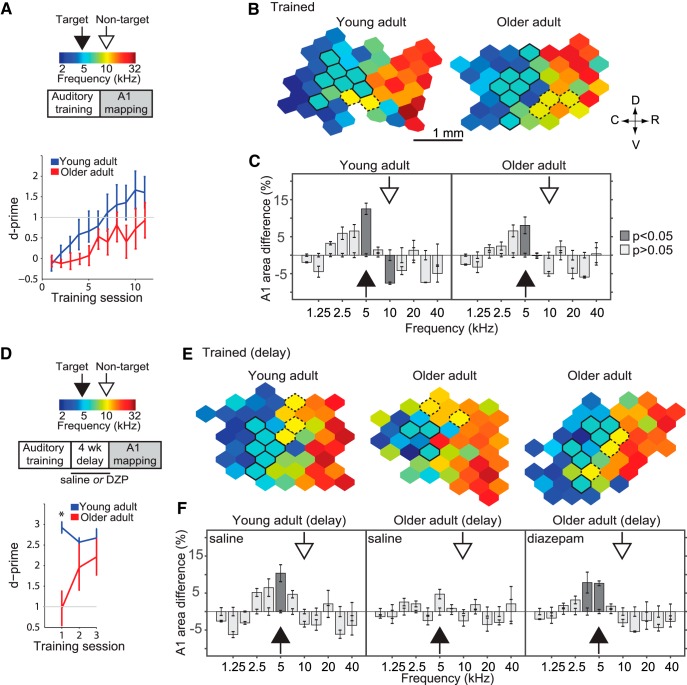
Aging and decay of training-induced A1 plasticity. Young and old adult rats were trained on a two-tone discrimination task (target tone: 10 kHz, nontarget: 5 kHz). ***A***, Top: Experimental protocol. Bottom: Older adult rats needed on average more training sessions to reach criterion than young adult rats (D-prime ≥1; YA no. of sessions = 8.4 ± 1.1; OA = 11.9 ± 1.4, *p* = 0.03). ***B***, Representative A1 characteristic frequency (CF) maps from trained young (left) and old (right) adult rats. Bolded polygons have a CF at the target tone ±0.3 octaves. Hatched polygons have a CF at the nontarget tone ±0.3 octaves. ***C***, Difference in frequency tuning between naïve and exposed rats expressed as A1 percentage area and separated by CF. The full arrows point to the target frequency; the hatched arrows points to the nontarget frequency. **D**, Top: To determine the persistence of learning and training-induced A1 map plasticity, a subgroup of YA-T and two subgroups of OA-T rats were subjected to a 4-week delay after reaching criterion, followed by behavioral re-assessment and A1 mapping. Bottom: From the first session of the reassessment onwards, young adult rats performed above criterion, while old adult rats performed above criterion from the second session onwards. ***E***, Representative A1 characteristic frequency (CF) maps from trained rats that received daily sham (saline) or diazepam (DZP) injections during the delay period. ***F***, Difference in A1 area tuned to various frequencies between each experimental group and untrained age-matched controls. YA-T group: *n* = 4, recorded sites = 212; OA-T: *n* = 4, recorded sites = 209; YA-T_delay_: *n* = 4; recorded sites = 192; OA-T_delay_: *n* = 4; recorded sites = 203; OA-T_delay(DZP)_: *n* = 4; recorded sites = 189. Values shown are mean ± SEM, *t* test, two-way ANOVA with Tukey–Kramer correction.

10.1523/ENEURO.0051-18.2018.f3-1 Figure 3-1 Summary of adaptation in response to repetitive tones. ***A***, Cumulative distribution plot of responses to repetitive tones (slope of normalized response rate to the standard tone) for all experimental groups (see Fig. 3*B* and Fig. 4*B*). ***B***, Reduced adaptation to repetitive tones (standard, circles) in immature (I) and old adult rats relative to young adults (YA). Adaptation was restored with the local administration of midazolam (asymptote of normalized response rate to standard tone; one-way ANOVA corrected for multiple comparisons with Tukey *post hoc* test, *p* = 3.8 × 10^–8^, *F*(4,1269) = 10.21; YA: 0.31 ± 0.019; I: 0.44 ± 0.017, *p* = 1.24 × 10^–5^, relative to YA; I-MDZ: 0.28 ± 0.015, *p* = 1.20 × 10^–5^, relative to I; OA: 0.43 ± 0.032, *p* = 5.9 × 10^–4^, relative to YA; OA-MDZ: 0.34 ± 0.027, *p* = 0.018, relative to OA). No significant differences in the overall magnitude of responses to oddballs (circles) was found between groups (asymptote of normalized response rate to oddball tones; one-way ANOVA, *p* = 0.29, *F*(4,1269) = 1.24). Both immature and aged groups showed a diminished response gap between standards and oddballs (height of gray vertical lines). This gap improved with the local administration of midazolam for immature but not old adult rats (asymptote difference between oddballs and standard; one-way ANOVA, *p* = 0.004, *F*(4,1269) = 3.84; YA, 0.30 ± 0.033; I, 0.19 ± 0.026, *p* = 0.057, relative to YA; OA, 0.15 ± 0.035, *p* = 0.0172, relative to YA; I-MDZ, 0.32 ± 0.024, *p* = 0.015 relative to I; OA-MDZ, 0.19 ± 0.041, *p* = 0.92, relative to OA; corrected for multiple comparisons). Immature group: *n* = 8, recorded sites = 376; YA: *n* = 4, recorded sites = 205; OA: *n* = 4, recorded sites = 192. YA group: *n* = 4, recorded sites = 205; I: *n* = 8, recorded sites = 376; OA: *n* = 4, recorded sites = 192; I-MDZ: *n* = 8, recorded sites = 346; OA-MDZ: *n* = 4, recorded sites = 155. Values shown are mean ± SEM. *p < 0.05. Download Figure 3-1, EPS file.

As expected, we found a progressive decrease in A1 neuron responses to repetitive tones in younger adults (median slope of normalized response rate: YA, –0.14 ± 0.03 × 10^−4^, number of recorded cortical sites = 205) but an increase in responses to repetitive tones in the immature and old adult groups relative to YA (median slope of normalized response rate: I, 0.09 ± 0.03 × 10^−4^, *p* = 4.1 × 10^−5^, *z* = –4.099^i^, number of recorded cortical sites = 376; OA, 0.11 ± 0.02 × 10^−4^, *p* = 0.0014, *z* = –3.187^j^, number of recorded cortical sites = 192; Wilcoxon rank-sum test; Fig. 3*B*, *C*).

Tuning stability in the same A1 neurons was examined by interspersing five oddball (low-probability) tones covering the hearing range during the repetitive (high-probability) tone presentation (see Methods). Using this method, coarse tuning curves could be constructed over two time intervals during the tone train exposure (T1, from 60 to 120 s; T2, from 400 to 460 s). On average, A1 neurons in the immature and old adult groups exhibited a significant increase in response to the high-probability tone from T1 to T2, while the opposite was seen in the young adult group (change in normalized firing rate, T2 minus T1: YA, –0.19 ± 0.05; I, 0.18 ± 0.04, *p* < 0.001, *t*(579) = 5.64^k^, relative to YA; OA, 0.15 ± 0.09 *p* = 9 × 10^−4^, *t*(395) = 3.35^l^, relative to YA; *t* test). Interestingly, the sum of responses to high and low probability tones remained constant in the immature and young adult groups, while it increased for the old adult group (difference in mean area under the curve between T1 and T2: I, 6.2 ± 3.3, *p* = 0.45, *t*(750) = 0.75^m^; YA, 4.78 ± 5, *p* = 0.52, *t*(408) = 0.64^n^; OA, 13.83 ± 2.71, *p* = 0.011, *t*(383) = 2.55^o^; paired *t* test; Fig. 3*D*, *E*).

In immature rats, short periods of auditory stimulation readily modify frequency tuning in A1, likely because of a disrupted E/I balance following the onset of hearing (Dorrn et al., 2010). Additionally, considering that GABA_A_-mediated inhibition regulates SSA (Duque et al., 2014), we hypothesized that transiently increasing inhibitory tone would improve adaptation in both the immature and aged A1. To test this possibility, we administered the short-acting GABA_A_ agonist midazolam during the presentation of the same repetitive stimulus. Given the different time scales between our adaptation (10 min) and passive exposure (1–2 weeks) experiments, we decided to use midazolam as opposed to the long-acting GABA_A_ agonist diazepam. Direct application of midazolam (1 μg/µl at a rate of 0.5 μl/min) to the cortex resulted on average in the progressive suppression of A1 responses to repetitive tones in the immature and older groups (median slope of normalized response rate: I, –0.13 ± 0.02 × 10^−4^, *p* = 1.1 × 10^−5^, *z* = –4.4^p^, number of recorded cortical sites = 346; OA, –0.01 ± 0.04 × 10^−4^, *p* = 0.013, *z* = –2.46^q^, number of recorded cortical sites = 155; Wilcoxon rank-sum test; Fig. 3*F*, *G* and Fig. 3-1*A*). It also resulted in a significant decrease in response to the high-probability tone in these groups (change in normalized firing rate in response to the standard tone, T2 minus T1; I: –0.12 ± 0.04, *p* < 0.001, *t*(720) = 5.29^r^; OA: –0.09 ± 0.06, *p* = 0.03, *t*(345) = 2.1^s^; *t* test). The overall response to the standard-oddball stimulus remained constant from T1 to T2 for both groups (difference in mean area under the curve between T1 and T2: I, 6.08 ± 4.55, *p* = 0.39, *t*(690) = 0.86^t^; OA, 1.63 ± 5.52, *p* = 0.94, *t*(308) = 0.08^u^; *t* test; Fig. 3*H*, *I*). A summary of A1 responses to repetitive tones and oddballs is provided in Fig. 3-1*B*.

### Impact of aging and dysregulated plasticity on auditory learning

Our results using passive sound exposure over different time scales suggest that age-related loss of inhibition could return the cortex into a state of instability where sensory representations are continuously distorted by nonspecific passive experience. If the deleterious effects of age-related loss of inhibition observed on passive experience extend to goal-oriented behavior, it is conceivable that reduced inhibition might contribute to make learning slower, harder, and more susceptible to decay, as has been clinically observed in older patients (Boyke et al., 2008; Lustig et al., 2009). To examine the impact of age on the retention of training-related plastic changes in A1, we compared the performance of young (*n* = 8) and older (*n* = 12) adult rats on an auditory discrimination task and then measured training-induced A1 changes at the end of training and after a 4-week delay. Both groups were trained on a two-tone discrimination task (target tone: 5 kHz, non-target tone: 10 kHz). Training ended once the rats’ discrimination reached a sustained value of D-prime (d′) ≥1 for two consecutive days; Fig. 4*A*, top). Older rats required on average more training sessions to reach criterion than younger adults (YA: 8.4 ± 0.8 sessions; OA: 11.9 ± 1.1, *p* = 0.032, *t*(18) = 2.32^v^; *t* test; Fig. 4*A*, bottom). At the end of training, A1 CF maps were obtained from a subgroup of young (YA-T, *n* = 4) and a subgroup of old adult rats (OA-T, *n* = 4; Fig. 4*B*). Two-way analysis of variance revealed a significant effect of training × frequency bin for both YA-T and OA-T groups (*F*(11,72) = 13.42, *p* < 0.001^w^; *F*(11,72) = 6.57, *p* < 0.001^x^; respectively). Compared to age-matched controls, both groups exhibited an increase in the number of neurons tuned to the target tone by the end of the training period (average % difference in the proportion of recording sites tuned within ½ octave of exposure tone, relative to control: YA-T: 12.25 ± 1.5% increase, *p* = 0.029^w^; OA-T: 8.06 ± 2.25% increase, *p* = 0.004^x^, corrected with Tukey–Kramer test; Fig. 4*C*). We also found, as previously reported (Voss et al., 2016), that the nontarget frequency was underrepresented in the trained YA but not in the OA group (YA-T: 11.54 ± 3.5% decrease, *p* = 0.018^w^; OA-T: 6.7 ± 2.2% decrease, *p* = 0.41^x^, corrected with Tukey–Kramer test; Fig. 4*C*).

To determine the retention of learning and persistence of training-related A1 retuning, we characterized trained younger (YA-T_delay_, *n* = 4) and older (OA-T_delay_, *n* = 4) rats after a 4-week delay period following completion of training (Fig. 4*D*, top). On average, younger rats maintained a significantly better performance than older when resuming training (YA-T_delay_: d′ = 2.8 ± 0.12; OA-T_delay_: d′ = 0.96 ± 0.48, *p* = 0.002 *t*(6) = 5.02^y^; Fig. 4*D*, bottom). A1 CF maps were reconstructed in another group of younger and older rats after the delay period (Fig. 4*E*). In these we found that the target tone representation in A1 had persisted in the younger but not older group (YA-T_delay_: *F*(11,72) = 6.68, *p* < 0.001, two-way ANOVA; 5 kHz: 10.37 ± 2.3% increase, *p* = 0.01^z^, corrected with Tukey–Kramer test; OA-T_delay_: *F*(11,72) = 1.41, *p* = 0.18^ab^, two-way ANOVA; 5 kHz: 4.72 ± 2.1% increase; Fig. 4*F*). Finally, to test whether pharmacologically increasing GABA inhibition would improve the retention of training-induced plastic changes, we treated a subgroup (*n* = 4) of older rats with diazepam (1 mg/kg i.p., twice a day) during the delay period post-training. A1 mapping in this group revealed a persistent target tone overrepresentation not significantly different from what had been observed immediately following training (*F*(11,72) = 5.42, *p* < 0.001, two-way ANOVA; 5 kHz: 7.64 ± 2.8% increase, *p* = 0.022^ac^, corrected with Tukey–Kramer test; Fig. 4*F*).

### Tonic GABAergic inhibition is reduced in the aged A1

**Figure 5.**
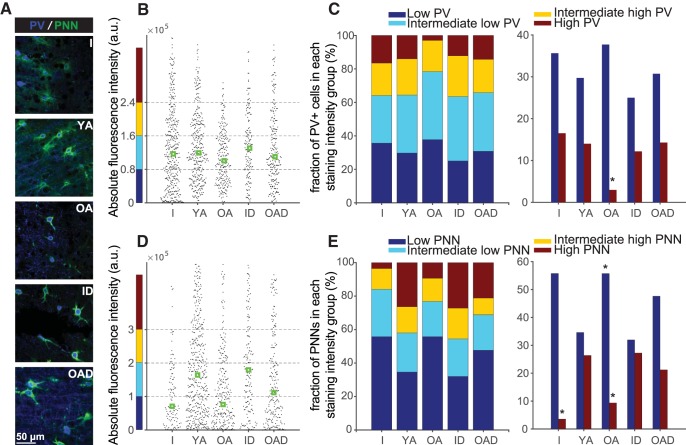
Impact of age on structural inhibitory elements in the auditory cortex. ***A***, High-power microphotographs of representative sections immunolabeled for perineuronal nets (PNN) and parvalbumin (PV) from immature (I), young adult (YA), old adult, immature + diazepam treatment (IA), and old adult + diazepam treatment (OAD) rats. ***B***, ***D***, Group fluorescence optical density for (***B***) PV and (***D***) PNN staining for each age group (all cortical layers; green boxes represent median values). ***C***, ***E***, Distribution of (***C***) PV cell and (***E***) PNN intensity staining for each age group. Fig. 5-1 compares A1 GABA concentration between YA and OA rats. Cell count per field for different neuronal types and age groups are detailed in Fig. 5-2. Fig. 5-3 shows representative micrographs of PV- and SST-positive cells. A summary of the cumulative distribution of staining intensity and interindividual variability for all groups is provided in Fig. 5-4. Number of hemispheres examined: I = 12, YA = 12, OA = 12, ID = 6, OAD = 6; total cell count per group: I = 418, YA = 343, OA = 236, ID = 156, OAD = 231. Values shown are mean ± SEM. *p < 0.05 relative to YA; Kruskal–Wallis test, corrected for multiple comparisons using Tukey–Kramer test.

To study the anatomic correlates of frequency tuning instability and impaired training performance, we sampled GABA concentration using microdialysis and quantified PV/PNN expression in A1 through immunohistochemistry. As documented in previous research (Morrison and Baxter, 2012; Rozycka and Liguz-Lecznar, 2017), we found that GABA concentration in A1 interstitial fluid was 25% lower in older adult rats (OA, 24 months old, *n* = 4) than in young adult controls (YA, 6 months old, *n* = 4) when measured in silence (YA, 100 ± 7.9%; OA, 75.2 ± 5.8% relative to YA; *p* = 0.04, *t*(6) = 2.53^ad^, *t* test; Fig. 5-1*A*). This difference was more pronounced during continuous sound presentation (see Methods). In the latter experimental condition, a relative reduction close to 40% was noted (YA-stim, 124 ± 7.7%; OA-stim, 88.2 ± 6.0% relative to YA-stim; *p* = 0.01, *t*(6) = 3.66^ae^, *t* test; Fig. 5-1*B*).

### Impact of age on perineuronal nets and PV^+^ neurons in A1

PV- and SST-positive cells constitute the two largest interneuron subpopulations throughout the cortex. In particular, PV^+^ neurons and associated PNNs are important regulators of experience-dependent plasticity throughout life (Caroni et al., 2012; Wang and Fawcett, 2012). Reduced cortical staining of PV and PNN are both associated with cortical immaturity and increased instability of cortical representations (Pizzorusso et al., 2002; McRae et al., 2007; Wang and Fawcett, 2012; Donato et al., 2013).

To assess whether age-related representational instability would be paralleled by a reduction in these plasticity-regulating structural elements, we first characterized the expression of the main interneuron subpopulations in the context of total cell counts for the three age groups—immature, young adult, and older adult—included in the present study (see Fig. 5-2 and Fig. 5-3 for cell counts and representative micrographs of interneurons, respectively). This analysis confirmed previous research showing a decrease in PV^+^ and SST^+^ cell counts associated with aging (Ouda et al., 2008; Ouellet and de Villers-Sidani, 2014). We then examined PV and PNN staining intensity in our different experimental groups (I, *n* = 6; YA, *n* = 6; OA, *n* = 6; ID, *n* = 3; OAD, *n* = 3; Fig. 5*A*). In line with previous reports (Hilbig et al., 2002; Ouda et al., 2008), we found decreased PV staining intensity with aging, which was recovered with 2-week-long diazepam treatment [median staining intensity ± MAD per PV^+^ cell, arbitrary confocal units (au) × 10^5^; *H*(4) = 14.52, *p* = 0.0058, Kruskal–Wallis test; I: 1.17 ± 0.85, *p* = 0.52, relative to YA; YA: 1.19 ± 0.69; OA: 1 ± 0.53, *p* = 0.011, relative to YA; ID: 1.3 ± 0.66, *p* = 0.96, relative to YA; OAD: 1.1 ± 0.76, *p* = 0.97^af^, relative to YA; Tukey–Kramer test; Fig. 5*B* and Fig. 5-4*A*]. PNN staining intensity, in contrast, showed a more contrasting lifetime trajectory, increasing from immature to young adult age and then reversing course with aging (Fig. 5*D*). Like our findings on PV^+^ cells, diazepam treatment resulted in recovery of PNN intensity staining for immature and older adult rats (median staining intensity per PNN, au × 10^5^; *H*(4) = 83.97, *p* < 0.0001, Kruskal–Wallis test; I: 0.7 ± 0.79, *p* < 0.0001, relative to YA; YA: 1.64 ± 1.37; OA: 0.76 ± 1.04, *p* < 0.001, relative to YA; ID: 1.79 ± 1.39, *p* = 0.96, relative to YA; OAD: 1.11 ± 1.33, *p* = 0.003^ag^, relative to YA; Tukey–Kramer test; Fig. 5*D* and Fig. 5-4*C*).

10.1523/ENEURO.0051-18.2018.f5-1 Figure 5-1 GABA concentration is reduced in the old adult A1. GABA concentration in A1 dialysate obtained (***A***) during silence and (***B***) during auditory stimulation from young adult (YA, *n* = 4) and old adult (OA, *n* = 4) rats. Values shown are mean ± SEM. *p < 0.05, **p < 0.01, *t* test. Download Figure 5-1, EPS file.

10.1523/ENEURO.0051-18.2018.f5-2 Figure 5-2 Interneuron cell count in A1 across the lifespan of the rat. Number of PV-, SST-, PNN-, GABA-, and Nissl-positive cells per field at P15 (*n* = 6), 6 months (*n* = 6), and 24 months (*n* = 6). Download Figure 5-2, DOCX file.

10.1523/ENEURO.0051-18.2018.f5-3 Figure 5-3 PV and SST expression in A1 interneurons. Representative high power confocal micrographs of (***A***) PV^+^ and (***B***) SST^+^ immunolabeled cells costained for GABA at the age intervals defined in Fig. 5-2. Download Figure 5-3, EPS file.

10.1523/ENEURO.0051-18.2018.f5-4 Figure 5-4 Restoration of PV^+^ and PNN staining intensity with diazepam. (***A***) Cumulative distribution plot and (***B***) individual variability of PV-labeling intensity for all experimental groups (see Fig. 5*B*). (***C***) Cumulative distribution plot and (***D***) individual variability of PNN-labeling intensity for all experimental groups (see Fig. 5*D*). Note that, although between-groups PV and PNN staining follow the same pattern; PV-staining data shows higher within-group variability. *p < 0.05, **p < 0.01, Kruskal–Wallis, corrected for multiple comparisons (Tukey–Kramer test). Download Figure 5-4, EPS file.

Further examination revealed that staining intensity of individual PV^+^ cells could be divided into four subgroups: low, intermediate low, intermediate high, and high intensity (Donato et al., 2013). We found a smaller proportion of high-intensity PV^+^ cells in older rats compared to young adults (*H*(4) = 13, *p* = 0.011^aj^, Kruskal–Wallis test; fraction of PV^+^ cells with low staining intensity and *p*-value relative to YA, per group: I_low_ = 16 ± 7.1%, *p* = 0.82; YA_low_ = 14 ± 3.2%; OA_low_ = 2.9 ± 0.4%, *p* = 0.005; ID_low_ = 12.1 ± 1.7%, *p* = 0.99; OAD_low_ = 14.2 ± 3.2%, *p* = 0.99^ah^; Tukey–Kramer test; Fig. 5*C* and Fig. 5-4*B*). A similar analysis was performed on PNNs, which could also be divided into four staining intensity groups. We found on average a higher proportion of low-intensity PNNs in aged rats compared to young adults (*H*(4) = 17.24, *p* = 0.0017, Kruskal–Wallis test; fraction of low-intensity PNNs and *p*-value relative to YA, per group: I_low_ = 55 ± 8.1%, *p* = 0.48; YA_low_ = 34 ± 2.2%; OA_low_ = 55 ± 1.7%, *p* = 0.04^ai^, Tukey–Kramer test) and a decrease in the high-intensity PNN subgroup in immature and aged rats compared to young adults (*H*(4) = 22.06, *p* < 0.001, Kruskal–Wallis test; fraction of high-intensity PNNs and *p*-value relative to YA, per group: I_high_ = 3.5 ± 1.7%, *p* = 0.004; YA_high_ = 26 ± 1.9%; OA_high_ = 9.3 ± 2.7%, *p* = 0.039^aj^; Tukey–Kramer test). Notably, following diazepam treatment, the proportion of low-intensity PNNs in older rats decreased, whereas the proportion of high-intensity PNNs in both immature and older rats increased, resulting in an intensity staining distribution that resembled that of the control (YA) group (fraction of PNN as a function of staining intensity and *p*-value relative to YA, per group: ID_low_ = 31.1 ± 1.9%, *p* = 0.8; OAD_low_ = 47 ± 3.1%, *p* = 0.99^ai^; ID_high_ = 27.2 ± 0.5%, *p* = 0.99; OAD_high_ = 21.2 ± 3.5%, *p* = 0.85^aj^; Tukey–Kramer test; Fig. 5*E* and Fig. 5-4*D*).

## Discussion

Our findings indicate that experience-dependent plasticity increases with aging following a natural reduction in cortical inhibition. Such increased plasticity may facilitate changes elicited by experience but also impair the brain’s capacity to crystallize such changes.

Brain aging is characterized by a down-regulation of cortical inhibition, which contributes to a range of functional deficits such as reduced selectivity of receptive fields, degraded temporal processing, heightened responses to noise, and reduced adaptation to repetitive stimuli (Turner et al., 2005; Hua et al., 2006; Caspary et al., 2008; Liguz-Lecznar et al., 2014; Schreiner and Polley, 2014).

What are the mechanisms of age-related reduction in inhibition? Recent findings suggest that reduced inhibition might not be a result of aging itself (Gourevitch et al., 2014). Young rats housed in a noisy auditory environment exhibit auditory perceptual deficits that mirror those observed in aging (Kamal et al., 2013; Gourevitch et al., 2014), alongside reduced GABA and interneuron expression (Zhou et al., 2011; Zhou and Merzenich, 2012). These impairments, however, are observed exclusively in rats exposed to continuous nonmodulated noise, but not after amplitude-modulated noise exposure (Thomas et al., 2018), suggesting that it is the lack of structured inputs—as opposed to noise per se—that drives maladaptive plasticity in the auditory cortex (Voss et al., 2017). It is therefore possible that age-related maladaptive plastic changes are a consequence of continuous, nonstructured “noisy” inputs, whether originating from the environment or resulting from conductive, sensorineural, or strial hearing loss (Jayakody et al., 2018). Prolonged exposure to distorted inputs might destabilize the activity of local neural circuits (Gourevitch et al., 2014) and trigger compensatory homeostatic changes (Burrone and Murthy, 2003; Dean et al., 2005; Turrigiano, 2011) that ultimately amplify excitatory inputs and reduce inhibition (Rothman et al., 2009; Tyagarajan et al., 2011).

The aforementioned studies strongly suggest that age-related anatomic and functional deficits can be modeled in noise-exposed young adult rats. Furthermore, rats exposed to nonstructured noise recover normal function when returned to their normal environment (Zhou and Merzenich, 2012; Kamal et al., 2013). Taken together, these observations suggest that perceptual deficits observed in the aged cortex have a significant activity-dependent component, rather than being purely age-related, and are thus at least partially reversible (Hilbig et al., 2002; Zhou and Merzenich, 2012; Liguz-Lecznar et al., 2014). For instance, GABA agonists increase selectivity of receptive fields in the primary visual cortex (Leventhal et al., 2003; Hua et al., 2006), classic conditioning enhances the expression of GABAergic markers in the barrel cortex (Liguz-Lecznar et al., 2014), and operant conditioning results in increased PV expression in A1 (de Villers-Sidani et al., 2010).

Functional deficits in the aged A1 include slowed and incomplete suppression of background distractors, which further impairs the detection of novel stimuli (de Villers-Sidani et al., 2010; Mishra et al., 2014). In the present study, this deficit was evident on a 10-min-long exposure to repetitive tones (Fig. 3*B–E*). We found impaired adaptation and tuning instability in the aged A1, whereas increasing inhibition with a short-acting GABAA agonist improved adaptation and reversed the tendency of aged A1 neurons to increase their tuning to the repetitive tone (Fig. 3*F–I*). Although there might be differences in the physiologic response to anesthesia between aged and adult animals, it should be noted that SSA is a property found in A1 and subcortical auditory nuclei that is minimally affected by anesthesia (Richardson et al., 2013; Duque and Malmierca, 2015).

Tuning instability was further confirmed by the fact that a short 1-week pip tone exposure sufficed to produce an overrepresentation of the exposure tone in older rats, as previously seen in immature rats (Fig. 1). However, this increased plasticity in the aged auditory cortex does not seem to be limited to a short time window, as is the case with the CP. In the present study, immature rats exposed successively to two different pure tones exhibited plasticity in response to the first tone, most likely because only the first tone exposure overlapped with the CP (Fig. 2*A*). Whereas a rapid and sustained increase in inhibition (Fagiolini and Hensch, 2000; Iwai et al., 2003; Hensch, 2005) ends the CP and prevents additional alterations due to passive sound exposure, a subsequent 1-week exposure to a different tone resulted in the overrepresentation of the latter tone in aged rats. Interestingly, boosting GABA inhibition consolidated frequency tuning representation and made the aging A1 again resistant to further alterations, thus “closing” this period of maladaptive increased plasticity (Fig. 2C). Follow-up studies may want to rule out the possibility, although unlikely, that diazepam selectively affects the processing of frequency tones in the 10-kHz range by presenting a lower frequency tone (e.g. 5 kHz) before the 10-kHz tone during diazepam treatment. Taken together, these findings suggest that the aging A1 appears to be in a permanent state of heightened plasticity to levels akin to those observed during early development.

The slower rate of learning in aged rats supports previous findings showing that age-related cortical processing deficits contribute to degraded behavioral performance (Barnes et al., 1997; Gazzaley et al., 2005; Samson and Barnes, 2013; Fig. 4*A–C*). According to the map expansion-renormalization model, initial sensory map expansion is necessary for discrimination learning (Takahashi et al., 2010; Reed et al., 2011). However, once subjects become experts at a task and reach a plateau in performance, their maps return to their previous state (Reed et al., 2011). In the present study, training was suspended before rats reached this plateau, and both groups exhibited typical training-induced map changes (Blake et al., 2006; Polley et al., 2006; Zhou et al., 2010). Learning becomes more susceptible to decay with aging (Lustig et al., 2009), which was evident after a 1-month delay period (Fig. 4D-F). Interestingly, training-induced map changes were preserved in the old rats treated with diazepam during the delay period between end of training and cortical mapping. Although map expansion was still present after this relatively short delay period, we did not measure the behavioral implications or the extent of this persistence beyond 1-month follow-up. Further studies will be necessary to fully understand the behavioral relevance of sustained map plasticity for learning.

Our findings of reduced PV and SST expression support numerous reports of reduced interneuron cell counts associated with aging (Rozycka and Liguz-Lecznar, 2017), suggesting that inhibitory deficits may be related to the dysfunction of specific interneuron cell subtypes (Cha et al., 1997; Ouda et al., 2008; Fish et al., 2013). Recent research, however, has focused on PV expression as a proxy for cellular function and has shown that cortical PV staining intensity is tightly correlated with the degree of experience-dependent plasticity (Zhou et al., 2011; Caroni, 2015). As a case in point, recent studies by Donato et al. (2013, 2015) demonstrate the impact of reduced PV staining on cell function. High-intensity PV^+^ cells are found on completion of learning and immediately after fear conditioning, situations in which stable, long-lasting sensory representations are warranted (Donato et al., 2013). Conversely, low-intensity PV^+^ cells are abundant during learning and following environmental enrichment, situations in which a more flexible cortical network is needed. Similarly, interventions that delay cortical maturation during early development (Chang and Merzenich, 2003; de Villers-Sidani et al., 2008) and those that impair auditory processing during adulthood (Martin del Campo et al., 2012; Zhou and Merzenich, 2012) result in decreased PV staining and increased plasticity. In line with these observations, we found a moderate increase in the low-PV fraction in the immature and aged A1, the age groups that showed increased experience-dependent plasticity. Moreover, the high-PV fraction was significantly diminished in the aged (Fig. 5*B*, *C*), which could account for the inadequate consolidation of newly formed sensory representations (Caroni et al., 2012; Donato et al., 2013).

PNNs are extracellular matrix deposits produced jointly by neurons and astrocytes, particularly around PV^+^ cells (McRae et al., 2007; Nakamura et al., 2009), forming both a structural and functional barrier that limits plasticity (Pizzorusso et al., 2002; Berardi et al., 2004; Wang and Fawcett, 2012). We found age-related changes in PNNs that mirrored those documented for PV^+^ cells; namely, a lower average staining density of PNNs in the extremes of life, characterized by an increase in the low-PNN fraction and a decrease in the high-PNN fraction. Interestingly, while age-related PNN intensity differences were more striking than those observed for PV^+^ cells, diazepam treatment in both cases resulted in a redistribution of the low- and high-intensity subgroups in immature and older adult rats toward values that resembled those of the young adult group (Fig. 5D, E). The disparity in histology results between PV^+^ cells and associated PNNs in immature rats supports the idea that PV^+^ cell development predates PNN assembly (Baker et al., 2017), suggesting that adequate PV^+^ cell functioning is required for PNN formation (Yamada et al., 2015; Quattromani et al., 2017).

The present study contributes to the understanding of how plasticity is regulated in the aged brain. Whereas previous studies have shown that GABAergic inhibition declines with age (Leventhal et al., 2003; Caspary et al., 2008; Liguz-Lecznar et al., 2014) and that passive sound exposure can alter cortical response properties in adulthood (Norena et al., 2006; Pienkowski et al., 2011), our study is the first one to show that A1 experience-dependent plasticity increases with aging. Further targeted manipulations of GABAergic function will be necessary to pinpoint the exact mechanisms underlying this age-related dysregulation of plasticity and to understand whether altered excitatory neurotransmission during aging (Benali et al., 2008) also plays a role.

Our findings have the potential to inform future research in animal models and humans. Recent studies have shown that cortical interneurons gate critical period plasticity locally (Takesian et al., 2018) and are necessary for sustained behavioral performance in trained animals (Kuchibhotla et al., 2017), but long-term outcomes of manipulating inhibitory neurotransmission remain unknown. Although we used a systemic GABA agonist, a logical next step in animal research would be to modulate inhibitory neurotransmission locally during passive exposure or learning using optogenetics or DREADDs for acute or chronic interventions, respectively.

In the human research domain, our findings may be particularly relevant to studies that are currently underway and that have potential clinical applications. In the absence of region-selective drugs to modulate GABAergic neurotransmission, studies using noninvasive brain stimulation (NIBS) are exploring the effects of manipulating cortical E/I balance on learning in the elderly (Opie and Cirillo, 2017). For instance, Opie et al. (2017) used two modalities of transcranial magnetic stimulation to alter cortical excitability before a motor learning task but found no benefit in healthy aged volunteers. In contrast, a subsequent study using transcranial direct current stimulation found that increasing inhibition before testing, followed by decreasing inhibition during testing, resulted in greater skill improvement in older adults (Fujiyama et al., 2017). We posit that a follow-up experiment using NIBS could be used to test our hypotheses of the role of inhibition in the acquisition and retention of learning—specifically, to test whether reducing inhibition early during training increases plasticity and facilitates learning, and whether increasing inhibition after learning facilitates the crystallization of newly acquired skills.

**Figure 6.**
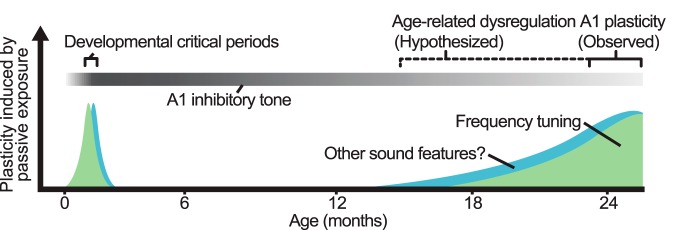
Proposed model of the impact of age on A1 plasticity. During periods of life characterized by a low inhibitory tone, passive exposure alters the A1 CF map. Plastic changes to the immature A1 are long lasting: as inhibition increases, the CP ends and sensory representations become stable. In contrast, plastic changes to the aged A1 are short-lived, as these cannot be consolidated due to a persistent low inhibitory tone.

Traditionally, aging has been regarded as a period of limited plasticity. However, our experiments suggest that this idea is unlikely to be correct in detail, as the aged brain is in some ways more plastic than the young adult brain. We propose that the inhibitory regulation of plasticity, rather than plasticity per se, is reduced in the aged brain (Fig. 6). Researchers and clinicians may build on this knowledge to develop rehabilitation strategies with at least two complementary objectives in mind: first, taking advantage of increased plasticity to enhance seniors’ functional recovery after neurologic injury, and second, regulating plasticity to preserve the benefits of rehabilitation and promote long-lasting recovery.
