# Supplementary material for: A Brain without Brakes: Reduced Inhibition Is Associated with Enhanced but Dysregulated Plasticity in the Aged Rat Auditory Cortex
Source: eNeuro. 2018 Sep 5;5(4):ENEURO.0051-18.2018. doi: 10.1523/ENEURO.0051-18.2018 (PMC6140119; doi:10.1523/ENEURO.0051-18.2018)
Supplement: Figure 5-2 — Interneuron cell count in A1 across the lifespan of the rat. Number of PV-, SST-, PNN-, GABA-, and Nissl-positive cells per field at P15 (n = 6), 6 months (n = 6), and 24 months (n = 6). Download Figure 5-2, DOCX file. [file sup_enu-eN-NWR-0051-18-s04.docx]

**Figure 5-2** Interneuron cell count in A1 across the lifespan of the rat.

|  |  | **Count by field (mean number of neurons)** | | | | |
| --- | --- | --- | --- | --- | --- | --- |
| **Group** | **Age** | **PV** | **Wisteria** | **SST** | **GABA** | **Nissl** |
|  |  |  |  |  |  |  |
| Immature | P15 | 4.635 | 1.548 | 1.100 | 13.357 | 107.889 |
| Young Adult | 6 mo. | 5.159 | 3.952 | 1.559 | 11.452 | 110.667 |
| Older Adult | 24 mo. | 3.690 | 3.579 | 0.825 | 8.254 | 105.711 |
